# GDF-15 is a potential candidate biomarker for an elevated risk of cardiotoxicity in breast cancer patients receiving neoadjuvant dual anti-HER2 therapy

**DOI:** 10.3389/fphar.2024.1396133

**Published:** 2024-05-17

**Authors:** Chunyu Tian, Hongxu Zhang, Jianping Liu, Mengze Xu, Lihui Ma

**Affiliations:** ^1^ Department of Breast Surgery, Affiliated Hospital of Chengde Medical University, Chengde, China; ^2^ School of Nursing, Chengde Medical University, Chengde, China

**Keywords:** GDF-15, biomarker, breast cancer, dual anti-HER2, cardiotoxicity

## Abstract

**Objective:**

Growth differentiation factor 15 (GDF-15) is a stress-responsive cytokine that regulates myocardial injury, cardiac overloading pressure, and inflammation and is related to the risk of cardiovascular diseases and events. The current study aimed to investigate the correlation of GDF-15 levels with clinical features, biochemical indices, and especially the risk of cardiotoxicity in breast cancer patients receiving neoadjuvant dual anti-HER2 therapy.

**Methods:**

A total of 103 HER2-positive breast cancer patients who underwent neoadjuvant dual anti-HER2 therapy (trastuzumab and pertuzumab plus chemotherapy) were included. Serum GDF-15 levels before neoadjuvant treatment were detected by enzyme-linked immunosorbent assay. Cardiotoxicity was evaluated during neoadjuvant therapy by referring to a decline of ≥10 percentage points in the left ventricular ejection fraction from baseline to an absolute level less than 50%.

**Results:**

GDF-15 exhibited a skewed distribution, with a median level of 714 (range: 207–1805) pg/mL. GDF-15 was positively correlated with age (*p* = 0.037), diabetes (*p* = 0.036), and the N-terminal pro-brain natriuretic peptide level (*p* = 0.013) and positively correlated with the total cholesterol level (*p* = 0.086) and troponin T level (*p* = 0.082), but these correlations were not statistically significant. A total of 6.8% of patients experienced cardiotoxicity during neoadjuvant therapy. By comparison, the GDF-15 level was greater in patients who experienced cardiotoxicity than in those who did not (*p* = 0.008). A subsequent receiver operating characteristic curve revealed that GDF-15 predicted cardiotoxicity risk, with an area under the curve of 0.803 (95% CI: 0.664–0.939). After multivariate adjustment, GDF-15 independently predicted a greater risk of cardiotoxicity (*p* = 0.020).

**Conclusion:**

GDF-15 is a candidate biomarker for increased risk of cardiotoxicity in breast cancer patients receiving neoadjuvant dual anti-HER2 therapy.

## 1 Introduction

Breast cancer is the most prevalent cancer type in women and contributes to 2.26 million newly diagnosed cancer cases worldwide, 0.30 million newly diagnosed cancer cases in America, and 0.31 million newly diagnosed cancer cases in China (Sung et al., 2021; Siegel et al., 2023; Zheng et al., 2023). The treatment options and prognoses of different subtypes of breast cancer vary (Gradishar et al., 2022); among these subtypes, the human epidermal growth factor receptor 2 (HER2)-positive subtype accounts for nearly 15%–25% of all breast cancer cases and has a relatively poor prognosis (Orrantia-Borunda et al., 2022). Encouragingly, since the development of trastuzumab, the first approved anti-HER2 drug, the prognosis of HER2-positive breast cancer patients has greatly improved (Swain et al., 2023). Currently, dual anti-HER2 therapy can further improve the outcomes in HER2-positive breast cancer patients with acceptable tolerance; therefore, dual anti-HER2 therapy is currently recommended as a first-line treatment globally and in China (Swain et al., 2020; Giordano et al., 2022; Li and Jiang, 2022).

Cardiotoxicity is an unavoidable concern in breast cancer patients receiving anti-HER2 therapy (Lin et al., 2021), and a recent review revealed that approximately 3%–7% of breast cancer patients receiving anti-HER2 therapy experience cardiac dysfunction of some form (Jerusalem et al., 2019). However, due to the different definitions of cardiotoxicity applied in distinct studies and the varied follow-up durations, the incidence of anti-HER2 therapy-induced cardiotoxicity varies widely (Mantarro et al., 2016; Schneeweiss et al., 2018; Swain et al., 2018; Al-Saleh et al., 2022). Since most of the cardiotoxicity induced by anti-HER2 therapy is subclinical, the most commonly used definition of cardiotoxicity in this condition is a decline of ≥10 percentage points in the left ventricular ejection fraction (LVEF) relative to baseline, along with an absolute decrease to less than 50% (Al-Saleh et al., 2022; Lu et al., 2023).

Investigations of biomarkers that recognize the risk of cardiotoxicity early in breast cancer patients receiving anti-HER2 therapy are ongoing (Ponde et al., 2018; Gherghe et al., 2022). Growth differentiation factor 15 (GDF-15), a stress-responsive cytokine in the transforming growth factor (TGF) family, can regulate myocardial injury, the overloading status of cardiac pressure, and inflammatory infiltration (Adela and Banerjee, 2015; Pence, 2022); moreover, GDF-15 is correlated with the risk of cardiovascular diseases such as cardiac hypertrophy, acute coronary syndrome, and heart failure (Havranek and Marek, 2021; May et al., 2021; Sawalha et al., 2023) and is capable of predicting the risk of cardiovascular events and death (Li et al., 2022; Xie et al., 2022). Interestingly, a study reported that GDF-15 estimates the risk of cardiotoxicity in breast cancer patients receiving adjuvant therapy with trastuzumab, doxorubicin, and axanes (Putt et al., 2015). However, the correlation between GDF-15 levels and cardiotoxicity risk in patients receiving neoadjuvant anti-HER2 therapy, especially dual anti-HER2 therapy, is still unclear.

Thus, this study aimed to investigate the correlation of GDF-15 levels with clinical features, biochemical indices, and especially its predictive value for the risk of cardiotoxicity in HER2-positive breast cancer patients receiving neoadjuvant dual anti-HER2 therapy.

## 2 Materials and methods

### 2.1 Patients

A total of 103 patients with HER2-positive breast cancer who received neoadjuvant dual anti-HER2 therapy between January 2021 and September 2023 were included in this study. The inclusion criteria were as follows: 1) had a pathological diagnosis of breast cancer; 2) were aged greater than or equal to 18 years; 3) had confirmed HER2 positivity; 4) received neoadjuvant docetaxel, carboplatin, trastuzumab, and pertuzumab (TCbHP) or docetaxel, trastuzumab, and pertuzumab (THP) treatment; 5) had available and complete data on cardiotoxicity during neoadjuvant treatment; 6) had accessible serum samples before neoadjuvant treatment; and 7) had available and complete data on clinical characteristics before neoadjuvant treatment. The exclusion criteria were as follows: 1) had distal metastasis; 2) LVEF at baseline was less than 50%; 3) had uncontrolled hypertension or diabetes; 4) had preexisting cardiac diseases such as coronary artery disease and heart failure; 5) had undergone prior oncologic therapy; and 6) lactating or pregnant women. The ethics committee approved the study. The patients or their families provided written informed consent.

### 2.2 Data retrieval

The following clinical characteristics were collected from the electronic medical systems for study analyses: 1) age; 2) menopausal status; 3) chronic diseases such as hypertension, hyperlipidemia, and diabetes; 4) disease-related characteristics such as the Eastern Cooperative Oncology Group Performance Status (ECOG PS), tumor node metastasis (TNM) stage, and the expression of human epidermal growth factor receptor 2 (HER2), hormone receptor (HR), and Ki67; 5) neoadjuvant treatment regimen; and 6) cardiovascular-related biochemical indices.

Patients received TCbHP or THP as neoadjuvant therapy based on their disease condition, willingness, and medical recommendation. Docetaxel (75 mg/m^2^) and carboplatin (area under the curve of 6 mg/mL/min) were administered intravenously every 3 weeks as part of the recommended TCbHP regimen. Docetaxel (80–100 mg/m^2^) was administered intravenously every 3 weeks as part of the recommended THP regimen. The loading dose of trastuzumab was 8 mg/kg, followed by intravenous administration of 6 mg/kg once every 3 weeks. Then, 840 mg of pertuzumab was used as the loading dose, and 420 mg was administered intravenously once every 3 weeks thereafter. The dose of each drug was adjusted according to the tolerance. Six cycles of neoadjuvant therapy were commonly administered (3 weeks per cycle).

### 2.3 Serum sample detection

Serum samples from patients collected before neoadjuvant treatment (at baseline) were used for study analyses, and serum GDF-15 levels were detected using a human GDF-15 enzyme-linked immunosorbent assay (ELISA) kit (Cat. No: E-EL-H0080, Elabscience Biotechnology Co., China).

### 2.4 Definition and evaluation of cardiotoxicity

The LVEF at baseline and after 2/4/6 cycles of treatment was collected. Specifically, the LVEF after cycles 1–3 was recorded as the LVEF after two cycles of treatment, the LVEF after cycles 3–5 was recorded as the LVEF after four cycles of treatment, and the LVEF after five or more cycles was recorded as the LVEF after six cycles of treatment. Then, cardiotoxicity was evaluated based on the LVEF data, which were defined as a decline of ≥10 percentage points in LVEF relative to the baseline, along with an absolute decrease to less than 50% (Al-Saleh et al., 2022; Lu et al., 2023).

### 2.5 Data categorization

For analyses regarding factors related to the risk of cardiotoxicity, GDF-15 was categorized into high and low using a cutoff value of 880.5 pg/mL (the best cutoff point, which was defined as the point on the ROC curve when the sum of sensitivity plus specificity reached the highest); age was categorized into elderly and nonelderly using a cutoff value of 60 years since people above 60 years of age were considered elderly people in China; tumor size was categorized into high and low using a cutoff value of 5 cm since it was an important boundary of tumor stage; Ki67 was categorized into high and low using a cutoff value of 30%, as commonly used in breast cancer studies; triglycerides (TG), total cholesterol (TC), low-density lipoprotein cholesterol (LDL-C), and high-density lipoprotein cholesterol (HDL-C) levels were categorized into abnormal and normal using cutoff values of 1.7, 5.2, 3.4, and 1.3 mmol/L, respectively, since they were the boundary settings in our hospital biochemical test; and TnT, NT-proBNP, and LVEF levels were categorized into high and low using cutoff values of their median values since their recognized cutoff values were not consistent in breast cancer patients.

### 2.6 Statistics

SPSS v26.0 (IBM, United States) was used for statistical analysis. The Mann‒Whitney *U* test and repeated measures one-way analysis of variance were used for comparison; Spearman’s rank correlation test was used for correlations. Receiver operating characteristic (ROC) curves were used to assess the ability of GDF-15 to distinguish breast cancer patients who experienced cardiotoxicity from those who did not. Logistic regression analysis was performed to assess cardiotoxicity, and the forward stepwise method was used to screen the independent factors, which were further estimated for performance by ROC curve analysis. A *p*-value of less than 0.05 was considered statistically significant.

## 3 Results

### 3.1 Characteristics description

The characteristics of the 107 eligible HER2-positive patients with breast cancer are shown in Table 1. The age of the patients was 51.1 ± 10.7 years. A total of 27.2%, 11.7%, and 11.7% of patients had hypertension, hyperlipidemia, and diabetes, respectively. The levels of TG, TC, LDL-C, HDL-C, TnT, and NT-proBNP were 1.1 (0.8–1.4) mmol/L, 3.7 (3.3–4.4) mmol/L, 1.9 (1.6–2.4) mmol/L, 1.2 (1.0–1.2) mmol/L, 6.0 (3.0–10.0) pg/mL, and 123.0 (91.0–148.0) pg/mL, respectively. In addition, 76.7% of patients received TCbHP neoadjuvant therapy, and 23.3% received THP neoadjuvant therapy.

**TABLE 1:** Clinical characteristics of breast cancer patients.

Items	Breast cancer patients (N = 103)
Age (years), mean ± SD	51.1 ± 10.7
Menopausal status, No. (%)	
Premenopausal	48 (46.6)
Postmenopausal	55 (53.4)
Hypertension, No. (%)	28 (27.2)
Hyperlipidemia, No. (%)	12 (11.7)
Diabetes, No. (%)	12 (11.7)
ECOG PS, No. (%)	
0	77 (74.8)
1	26 (25.2)
Tumor size (cm), mean ± SD	4.8 ± 1.3
T stage, no. (%)	
T2	71 (68.9)
T3	32 (31.1)
N stage, no. (%)	
N0	17 (16.5)
N1	50 (48.5)
N2	36 (35.0)
M stage, no. (%)	
M0	103 (100.0)
TNM stage, no. (%)	
IIA	5 (4.9)
IIB	51 (49.5)
IIIA	47 (45.6)
HER2 positivity, no. (%)	103 (100.0)
HER2 determination, no. (%)	
IHC++ plus FISH confirmation	19 (18.4)
IHC+++	84 (81.6)
HR positivity, no. (%)	64 (62.1)
Ki67 expression >30%, no. (%)	53 (51.5)
Regimen, no. (%)	
THP	24 (23.3)
TCbHP	79 (76.7)
TG (mmol/L), median (IQR)	1.1 (0.8–1.4)
TC (mmol/L), median (IQR)	3.7 (3.3–4.4)
LDL-C (mmol/L), median (IQR)	1.9 (1.6–2.4)
HDL-C (mmol/L), median (IQR)	1.2 (1.0–1.2)
TnT (pg/mL), median (IQR)	6.0 (3.0–10.0)
NT-proBNP (pg/mL), median (IQR)	123.0 (91.0–148.0)
LVEF (%), mean ± SD	62.4 ± 3.5

SD, standard deviation; ECOG PS, Eastern Cooperative Oncology Group Performance Status; TNM, tumor node metastasis; HER2, human epidermal growth factor receptor 2; IHC, immunohistochemistry; FISH, fluorescence *in situ* hybridization; HR, hormone receptor; THP, docetaxel, trastuzumab, and pertuzumab; TCbHP, docetaxel, carboplatin, trastuzumab, and pertuzumab; TG, triglycerides; IQR, interquartile range; TC, total cholesterol; LDL-C, low-density lipoprotein cholesterol; HDL-C, high-density lipoprotein cholesterol; TnT, troponin T; NT-proBNP, N-terminal pro-brain natriuretic peptide; LVEF, left ventricular ejection fraction.

### 3.2 GDF-15 distribution

**FIGURE 1:**
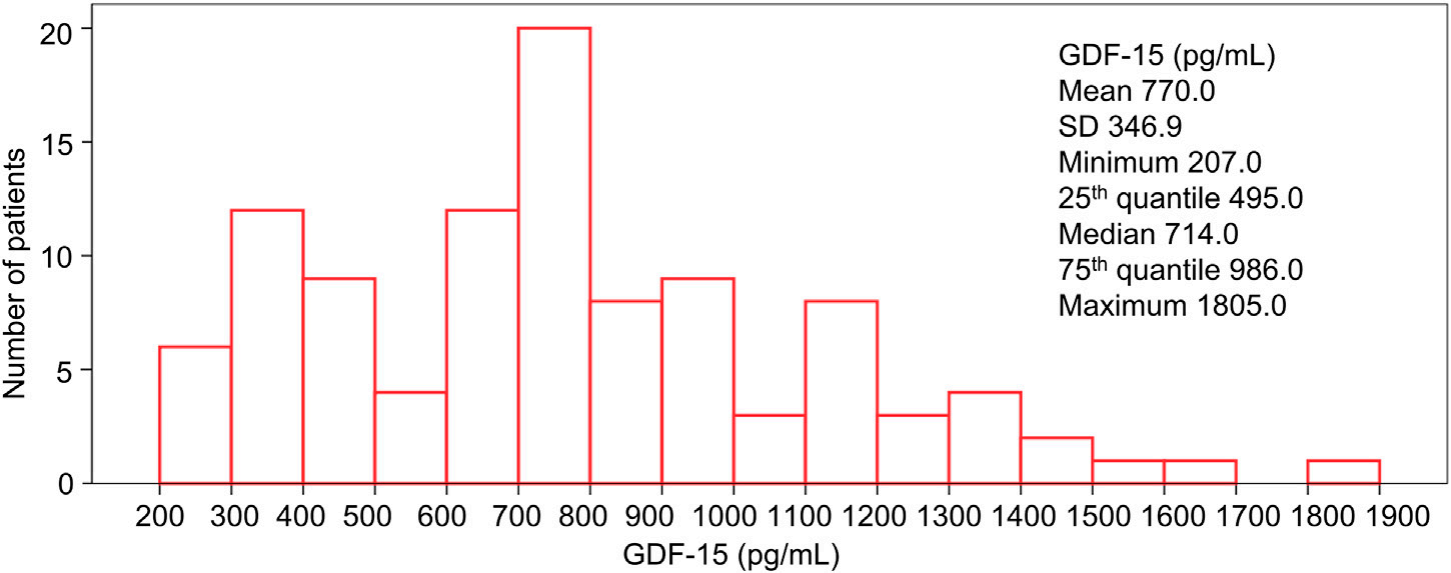
Distribution of GDF15 levels in HER2-positive breast cancer patients.

Generally, the GDF-15 level exhibited a skewed distribution in HER2-positive breast cancer patients (Figure 1). The median level of GDF-15 was 714.0 pg/mL; the minimum, 25th quantile, 75% quantile, and maximum levels of GDF-15 were 207.0, 495.0, 986.0, and 1,805.0 pg/mL, respectively.

### 3.3 Correlation of GDF-15 with clinical features and biochemical indices

GDF-15 levels were positively correlated with age (*p* = 0.037), diabetes (*p* = 0.036), and NT-proBNP levels (*p* = 0.013) in HER2-positive breast cancer patients and tended to be positively correlated with TC levels (*p* = 0.086) and TnT levels (*p* = 0.082), but the correlations were not statistically significant (Table 2). However, GDF-15 levels were not correlated with other clinical features, biochemical indices, or neoadjuvant therapy regimens.

**TABLE 2:** Correlation of GDF-15 levels with clinical characteristics.

Items	GDF-15 level (pg/mL) and median (IQR)	*r* value	*p*-value
Age	—	0.206	0.037
Menopausal status		—	0.218
Premenopausal	681.0 (487.5–936.0)		
Postmenopausal	757.0 (523.0–1,032.0)		
Hypertension		—	0.278
No	709.0 (444.0–947.0)		
Yes	758.0 (534.0–1,093.0)		
Hyperlipidemia		—	0.582
No	714.0 (485.0–967.0)		
Yes	783.5 (670.3–1,044.0)		
Diabetes		—	0.036
No	709.0 (473.0–947.0)		
Yes	932.0 (696.5–1,234.5)		
ECOG PS		—	0.215
0	712.0 (490.0–950.0)		
1	799.0 (511.3–1,122.5)		
Tumor size (cm)	—	0.055	0.581
T stage			0.195
T2	707.0 (417.0–947.0)		
T3	736.0 (545.3–1,071.5)		
N stage		—	0.271
N0	714.0 (490.0–905.5)		
N1	705.5 (492.5–952.0)		
N2	745.0 (490.5–1,159.8)		
TNM stage		—	0.106
IIA	773.0 (549.0–924.0)		
IIB	702.0 (444.0–921.0)		
IIIA	761.0 (564.0–1,166.0)		
HER2 determination		—	0.702
IHC++ plus FISH confirmation	714.0 (495.0–872.0)		
IHC+++	718.0 (490.5–1,020.5)		
HR positivity, no. (%)		—	0.338
Negative	704.0 (383.0–922.0)		
Positive	726.5 (534.0–989.0)		
Ki67 expression		—	0.514
≤30%	749.0 (479.8–1,000.5)		
>30%	704.0 (497.0–950.0)		
Regimen		—	0.845
THP	740.0 (534.0–940.8)		
TCbHP	714.0 (482.0–1,032.0)		
TG (mmol/L)	—	0.148	0.135
TC (mmol/L)	—	0.170	0.086
LDL-C (mmol/L)	—	0.156	0.116
HDL-C (mmol/L)	—	0.157	0.114
TnT (pg/mL)	—	0.172	0.082
NT-proBNP (pg/mL)	—	0.245	0.013

Correlations of two continuous variables were analyzed using Spearman’s rank correlation test and are shown with *r* and *p* values. The correlations of GDF-15 levels with N stage and TNM stage were analyzed using Spearman’s rank correlation test and are shown with medians (IQRs) and *p* values. The GDF-15 levels in patients with different categorical characteristics were analyzed using the Mann‒Whitney *U* test and are shown with medians (IQRs) and *p* values. GDF-15, growth differentiation factor 15; IQR, interquartile range; ECOG PS, Eastern Cooperative Oncology Group Performance Status; TNM, tumor node metastasis; HER2, human epidermal growth factor receptor 2; IHC, immunohistochemistry; FISH, fluorescence *in situ* hybridization; HR, hormone receptor; THP, docetaxel, trastuzumab, and pertuzumab; TCbHP, docetaxel, carboplatin, trastuzumab, and pertuzumab; TG, triglycerides; TC, total cholesterol; LDL-C, low-density lipoprotein cholesterol; HDL-C, high-density lipoprotein cholesterol; TnT, troponin T; NT-proBNP, N-terminal pro-brain natriuretic peptide.

### 3.4 Correlation of GDF-15 with LVEF

**FIGURE 2:**
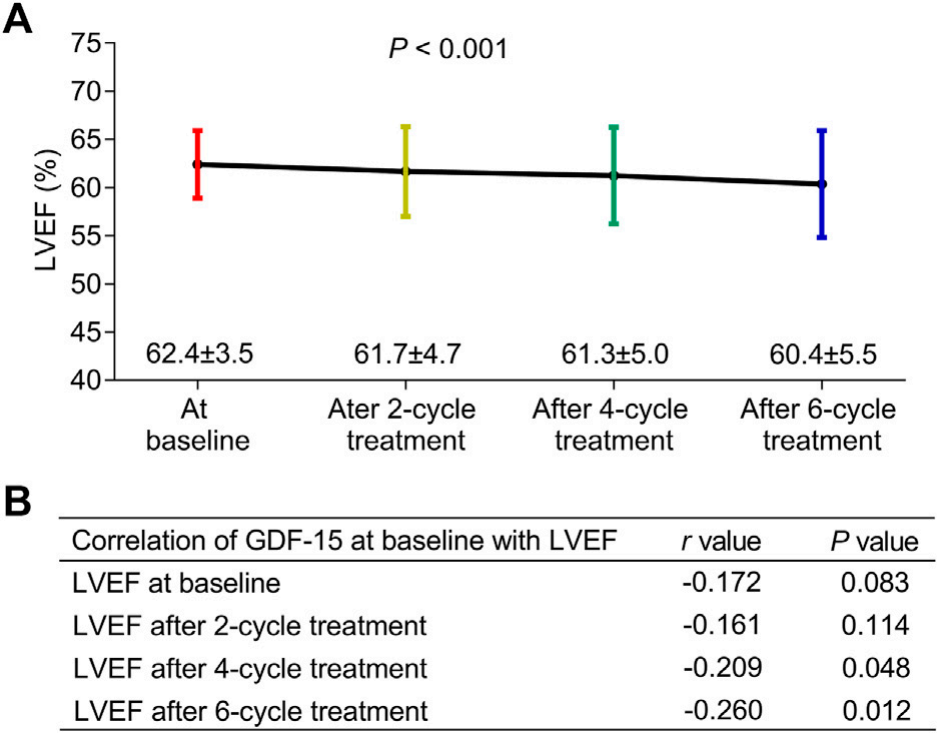
Correlation of GDF-15 levels with LEVF levels in HER2-positive breast cancer patients. The LVEF at the baseline, after two cycles of treatment, after four cycles of treatment, and after six cycles of treatment in HER2-positive breast cancer patients receiving neoadjuvant dual anti-HER2 therapy **(A)**. Correlation of the GDF-15 level (at baseline) with the LVEF at the baseline, after two cycles of treatment, after four cycles of treatment, and after six cycles of treatment in those patients **(B)**.

The LVEF gradually decreased during the entire neoadjuvant therapy period (*p* < 0.001) (Figure 2A); in detail, the LVEF decreased to 62.4% ± 3.5%, 61.7% ± 4.7%, 61.3% ± 5.0%, and 60.4% ± 5.5% at baseline, after two cycles of treatment, after four cycles of treatment, and after six cycles of treatment, respectively. In addition, the baseline GDF-15 level was negatively correlated with the LVEF after four cycles of treatment (*p* = 0.048) and after six cycles of treatment (*p* = 0.012) (Figure 2B).

### 3.5 Correlation of GDF-15 with cardiotoxicity risk

**FIGURE 3:**
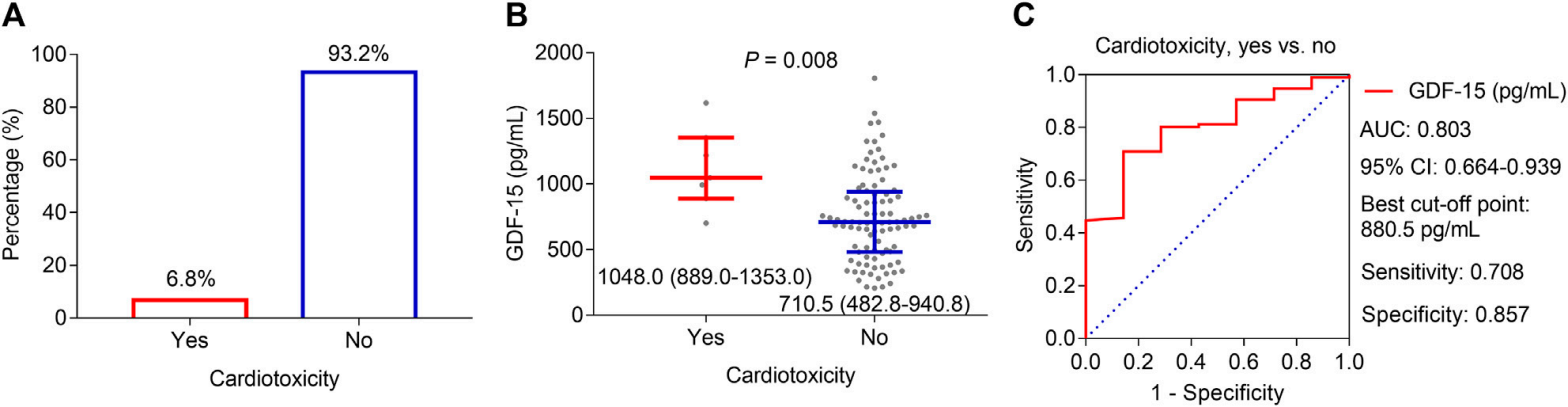
Correlation of GDF-15 levels with cardiotoxicity in HER2-positive breast cancer patients. Cardiotoxicity incidence in HER2-positive breast cancer patients receiving neoadjuvant dual anti-HER2 therapy **(A)**. Difference in GDF-15 levels between patients who experienced cardiotoxicity and those who did not **(B)**. ROC curve of the GDF-15 value for cardiotoxicity risk **(C)**.

During the period of neoadjuvant dual anti-HER2 therapy, 6.8% of patients experienced a decline of ≥10 percentage points in LVEF relative to the baseline and an absolute decrease to less than 50% simultaneously, which was defined as cardiotoxicity (Figure 3A). By comparison, the GDF-15 level was much greater in patients who experienced cardiotoxicity (median, interquartile range (IQR): 1,048.0, 889.0–1,353.0 pg/mL) than in those who did not (median, IQR: 710.5, 482.8–940.8 pg/mL) (*p* = 0.008, Figure 3B). Subsequent ROC curve analysis revealed that the GDF-15 level could predict the risk of cardiotoxicity, with an area under the curve (AUC) of 0.803 (95% CI: 0.664–0.939) (Figure 3C). In particular, when choosing the best cutoff point (880.5 pg/mL), the sensitivity and specificity were 70.8% and 85.7%, respectively.

### 3.6 Predictive model involving GDF-15 for cardiotoxicity risk

**FIGURE 4:**
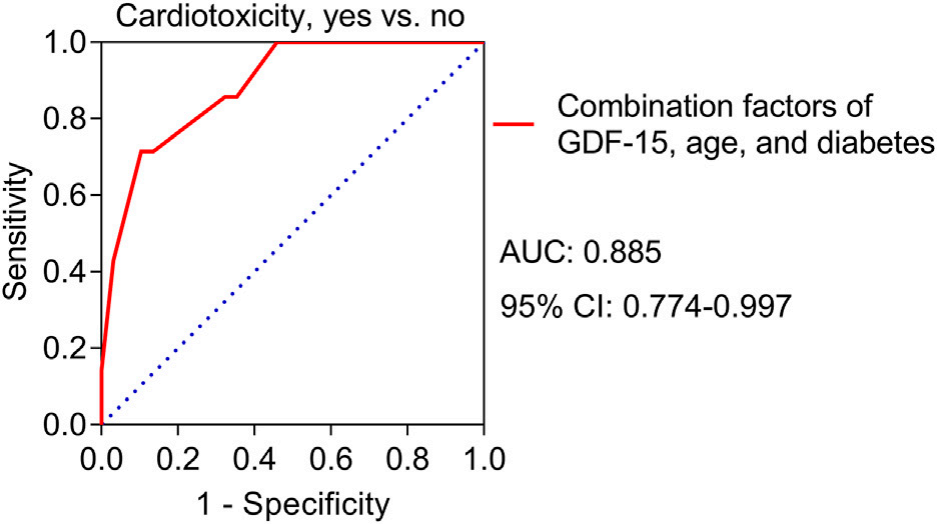
ROC curve for the value of the predictive model for cardiotoxicity risk in HER2-positive breast cancer patients receiving neoadjuvant dual anti-HER2 therapy.

Univariate logistic regression analyses revealed that high GDF-15 levels (*p* = 0.015) and diabetes (*p* = 0.018) were correlated with increased cardiotoxicity risk (Table 3) and high TnT levels (*p* = 0.081) and high NT-proBNP levels (*p* = 0.075) tended to be correlated with elevated cardiotoxicity risk but were not statistically significant. Multivariate logistic regression analyses further revealed that high GDF-15 levels (*p* = 0.020) and diabetes (*p* = 0.034) were independently correlated with increased cardiotoxicity risk; in addition, advanced age (*p* = 0.052) tended to be independently correlated with increased cardiotoxicity risk, but the correlation was not statistically significant. A predictive model involving high GDF-15 levels, diabetes status, and advanced age was constructed, and the ROC curve showed that the predictive model had good value in predicting the risk of cardiotoxicity, with an AUC of 0.885 (95% CI: 0.774–0.997) (Figure 4).

**TABLE 3:** Logistic regression analysis for cardiotoxicity.

Items	*p*-value	OR	95% CI
Univariate logistic regression models			
GDF-15 (pg/mL), high vs. low	0.015	14.571	1.677–126.637
Age (years), elderly vs. non-elderly	0.837	1.176	0.250–5.541
Menopausal status, postmenopausal vs. premenopausal	0.333	2.300	0.425–12.440
Hypertension, yes vs. no	0.344	2.130	0.445–10.185
Hyperlipidemia, yes vs. no	0.170	3.440	0.588–20.110
Diabetes, yes vs. no	0.018	7.250	1.397–37.629
ECOG PS, 1 vs. 0	0.279	2.380	0.496–11.426
Tumor size (cm), high vs. low	0.246	0.368	0.068–1.990
T stage, per stage	0.883	0.880	0.161–4.796
N stage, per stage	0.140	2.715	0.720–10.235
TNM stage, per stage	0.445	1.741	0.420–7.225
HER2 determination, IHC+++ vs. IHC++ plus FISH confirmation	0.770	1.385	0.157–12.226
HR positive, yes vs. no	0.602	1.568	0.289–8.501
Ki67 expression, high vs. low	0.287	2.500	0.462–13.521
Regimen, TCbHP vs. THP	0.565	1.890	0.216–16.528
TG (mmol/L), abnormal vs. normal	0.336	2.343	0.413–13.282
TC (mmol/L), abnormal vs. normal	0.956	1.064	0.118–9.568
LDL-C (mmol/L), abnormal vs. normal	0.261	3.833	0.369–39.865
HDL-C (mmol/L), abnormal vs. normal	0.530	0.577	0.104–3.216
TnT (pg/mL), high vs. low	0.081	6.800	0.788–58.647
NT-proBNP (pg/mL), high vs. low	0.075	7.091	0.822–61.163
LVEF at baseline (%), high vs. low	0.109	0.251	0.046–1.360
Forward stepwise multivariate logistic regression model			
GDF-15 (pg/mL), high vs. low	0.020	16.835	1.567–180.822
Age (years), elderly vs. non-elderly	0.052	6.491	0.984–42.809
Diabetes, yes vs. no	0.034	8.403	1.171–60.298

Continuous variables were cut into binary categorical variables with the following cutoff values: GDF-15, 880.5 pg/mL; age, 60 years; tumor size, 5 cm; Ki67 expression, 30%; TG, 1.7 mmol/L; TC, 5.2 mmol/L; LDL-C, 3.4 mmol/L; HDL-C, 1.3 mmol/L; TnT, median value (for the lack of clinical consensus cutoff values); NT-proBNP, median value (for the lack of clinical consensus cutoff values); and LVEF, median value (for all patients within the normal range). OR, odds ratio; CI, confidence interval; GDF-15, growth differentiation factor 15; ECOG PS, Eastern Cooperative Oncology Group Performance Status; TNM, tumor node metastasis; HER2, human epidermal growth factor receptor 2; IHC, immunohistochemistry; FISH, fluorescence *in situ* hybridization; HR, hormone receptor; TCbHP, docetaxel, carboplatin, trastuzumab, and pertuzumab; THP, docetaxel, trastuzumab, and pertuzumab; TG, triglycerides; TC, total cholesterol; LDL-C, low-density lipoprotein cholesterol; HDL-C, high-density lipoprotein cholesterol; TnT, troponin T; NT-proBNP, N-terminal pro-brain natriuretic peptide; LVEF, left ventricular ejection fraction.

## 4 Discussion

Dual anti-HER2 therapy has improved the outcomes of HER2-positive breast cancer patients, but at the same time, it may increase the risk of treatment-induced cardiotoxicity to some extent (Swain et al., 2020; Huang et al., 2022). A previous study reported that 8.6% of HER2-positive breast cancer patients undergoing dual anti-HER2 therapy experienced cardiotoxicity (Huang et al., 2022), and another study reported that the rate of cardiotoxicity was 2.0%–6.5% under dual anti-HER2 therapy (Swain et al., 2018). Interestingly, a recent large-scale study involving 4,769 HER2-positive breast cancer patients revealed that 3.3% of patients experienced cardiotoxicity during dual anti-HER2 therapy (de Azambuja et al., 2023). The current study revealed that 6.8% of HER2-positive breast cancer patients experienced cardiotoxicity during neoadjuvant dual anti-HER2 therapy, which was within the range of cardiotoxicity reported in previous studies (Mantarro et al., 2016; Schneeweiss et al., 2018; Swain et al., 2018; Al-Saleh et al., 2022; Huang et al., 2022; de Azambuja et al., 2023). However, the rate of cardiotoxicity in the current study was slightly greater than the average reported by previous studies. Several explanations were provided, which were as follows (Sung et al., 2021): the baseline LVEF was relatively lower in the current study (Siegel et al., 2023); the definition of cardiotoxicity differed among different studies, and the current study applied the definition of cardiotoxicity as a decline of ≥10 percentage points in LVEF relative to the baseline, along with an absolute decrease to less than 50% (Al-Saleh et al., 2022; Lu et al., 2023), which was relatively loose; therefore, the rate of identified cardiotoxicity was relatively greater.

GDF-15 has been applied as a biomarker for predicting the risk of cardiovascular diseases and cardiovascular events (Adela and Banerjee, 2015; Havranek and Marek, 2021; May et al., 2021; Li et al., 2022; Pence, 2022; Xie et al., 2022; Sawalha et al., 2023). For instance, GDF-15 is upregulated in patients with coronary artery disease (CAD) compared to non-CAD controls, yielding a diagnostic value with an AUC of 0.9 for CAD (Hassanzadeh Daloee et al., 2021); moreover, GDF-15 is greater in heart failure patients with ischemic heart disease than in controls without ischemic heart disease (Elsewify et al., 2022). In addition, GDF-15 predicts the risk of major adverse cardiovascular events in patients with diabetes, as reported by a meta-analysis involving nearly twenty thousand patients (Xie et al., 2022). Furthermore, a very interesting meta-analysis reports that GDF-15 is consistently useful for prognostic prediction of the risk of cardiovascular death and hospitalization for heart failure among different types of atherosclerotic cardiovascular diseases (Kato et al., 2023).

Regarding drug-induced cardiotoxicity in breast cancer patients, a study revealed that GDF-15 can predict the risk of cardiotoxicity in breast cancer patients receiving adjuvant trastuzumab-based anti-HER2 therapy (Putt et al., 2015). However, the predictive value of GDF-15 levels for the risk of cardiotoxicity induced by neoadjuvant dual anti-HER2 therapy has not been reported in HER2-positive breast cancer patients, which is clinically important. Our current study was built on the existing studies that report the value of GDF-15 quantification in predicting cardiotoxicity risk in breast cancer patients receiving trastuzumab-based treatment (Ky et al., 2014; Putt et al., 2015; Yu et al., 2018; Kirkham et al., 2022). The differences of our study from the mentioned studies included the following (Sung et al., 2021): they focused on trastuzumab-based treatment or doxorubicin-induced cardiotoxicity, while we focused on dual anti-HER2 therapy (Siegel et al., 2023); they focused on the adjuvant settings, while we focused on the neoadjuvant setting.

The present study revealed that in HER2-positive breast cancer patients receiving neoadjuvant dual anti-HER2 therapy, the GDF-15 level was greater in patients who experienced cardiotoxicity than in those who did not. Subsequent ROC curve analysis revealed that the GDF-15 level predicted the risk of cardiotoxicity, with an AUC of 0.803 (95% CI: 0.664–0.939), which is an inspiring finding. Furthermore, after adjustment by multivariate analysis, the GDF-15 level still independently estimated the risk of cardiotoxicity in those patients. These results indicated that GDF-15 could serve as a candidate biomarker for increased risk of cardiotoxicity induced by neoadjuvant dual anti-HER2 therapy. The explanations might be the close relationship of GDF-15 with myocardial injury, cardiac overloading pressure status, inflammation, etc. (Adela and Banerjee, 2015; Pence, 2022). Furthermore, the current study revealed that GDF-15 levels were positively correlated with age, diabetes status, and NT-proBNP levels in HER2-positive breast cancer patients, which might explain the predictive value of GDF-15 for cardiotoxicity risk to some extent. It is noteworthy that GDF-15 is an established biomarker of diabetes and cardiovascular disease. Therefore, it is uncertain if the association between baseline GDF-15 and the risk of cardiotoxicity would remain statistically significant when analyzing a cohort that does not have any pre-existing cardio-metabolic conditions. To investigate this issue, we screened out the patients without hypertension, hyperlipidemia, or diabetes in this current study and then analyzed the correlation between GDF-15 and cardiotoxicity. However, in the patients without hypertension, hyperlipidemia, or diabetes, the cardiotoxicity only occurred in one patient (1.6%); therefore, it cannot be analyzed. The one patient who experienced cardiotoxicity had a GDF-15 level of 889 pg/mL; the number was higher than the median level 703.5 pg/mL of those without cardiotoxicity in the cohort without hypertension, hyperlipidemia, or diabetes. These indicate that the control of risk factors (such as hypertension and diabetes) is sub-optimal for cardiotoxicity prevention during breast cancer treatment (Kaneko et al., 2023).

Unavoidably, some limitations existed in the current study. First, GDF-15 levels were detected at a single time point (at baseline, which was the time before initiation of neoadjuvant dual anti-HER therapy), and the variation in GDF-15 levels during or after neoadjuvant treatment was not investigated. Second, the source of blood GDF-15 was not investigated. Third, the relation between GDF-15 and dual anti-HER2 plus endocrine therapy-induced cardiotoxicity was not investigated in this study. Fourth, this was a single-center study, which might have biases; thus, further validation by a multiple-center study is needed. Fifth, the sample size was not large enough; moreover, a validation cohort in the future would be more desirable to make a firm conclusion.

Overall, GDF-15 serves as a candidate biomarker for predicting an increased risk of cardiotoxicity in breast cancer patients receiving neoadjuvant dual anti-HER2 therapy. However, more evidence is needed for validation to make a final conclusion.

## Data Availability

The original contributions presented in the study are included in the article/Supplementary Material; further inquiries can be directed to the corresponding author.
